# Cell Therapies in Cardiomyopathy: Current Status of Clinical Trials

**DOI:** 10.1155/2017/9404057

**Published:** 2017-01-17

**Authors:** Ming Hao, Richard Wang, Wen Wang

**Affiliations:** ^1^Cellular Biomedicine Group, 333 Guiping Road, Shanghai 200233, China; ^2^Cellular Biomedicine Group, 19925 Stevens Creek Blvd, Suite 100, Cupertino, CA 95014, USA

## Abstract

Because the human heart has limited potential for regeneration, the loss of cardiomyocytes during cardiac myopathy and ischaemic injury can result in heart failure and death. Stem cell therapy has emerged as a promising strategy for the treatment of dead myocardium, directly or indirectly, and seems to offer functional benefits to patients. The ideal candidate donor cell for myocardial reconstitution is a stem-like cell that can be easily obtained, has a robust proliferation capacity and a low risk of tumour formation and immune rejection, differentiates into functionally normal cardiomyocytes, and is suitable for minimally invasive clinical transplantation. The ultimate goal of cardiac repair is to regenerate functionally viable myocardium after myocardial infarction (MI) to prevent or heal heart failure. This review provides a comprehensive overview of treatment with stem-like cells in preclinical and clinical studies to assess the feasibility and efficacy of this novel therapeutic strategy in ischaemic cardiomyopathy.

## 1. Introduction

Ischaemic cardiomyopathy, which mainly results from the blockage of multiple coronary arteries, is the most common cause of early death in adults worldwide [1]. A myocardial infarction (MI) can kill approximately 25% of cardiomyocytes in only a few hours [2]. However, the adult human heart has limited potential for regeneration to repair the injury caused by MI. Over the past two decades, cardiac transplantation has been the only available cure for people who develop advanced heart failure [3].

Cardiac homeostasis has traditionally been considered to be static in the adult mammalian heart. This might seem perplexing because the heart is one of the least regenerative organs, and it possesses a relatively constant number of myocytes that are as old as the individual [4]. Even under the most ideal circumstances, when all therapeutic interventions are applied to preserve the remaining myocytes from death, a moderate rate of cellular apoptosis leads to the erosion of the myocardium over time. In this case, the onset of heart failure in the elderly appears to be inevitable.

Currently, remarkable progress has been made to demonstrate the presence of cycling cardiomyocytes in humans [5–7]. Radiocarbon birth dating has suggested that turnover rate in the endogenous adult human heart is approximately 1% per year, with approximately 45% of cardiomyocytes predicted to be renewed after birth [8]. Unfortunately, the injury from an acute MI cannot be reversed by resident cardiomyocyte proliferation during normal aging. Pulse-chase labelling has suggested that cardiac stem/precursor cells contribute to cardiomyocytes replenishment and regeneration after injury [9]. Therefore, the existence of cardiac stem-like cells promises a tantalizing approach to the treatment of ischaemic cardiomyopathy.

The ultimate goal of cardiac repair is to regenerate functionally viable myocardium after MI to prevent or heal heart failure. Conventional surgical interventions, such as coronary artery bypass graft (CABG) or percutaneous coronary intervention (PCI), are only able to restore heart function to a minor degree, with an improvement in the left ventricular ejection fraction (LVEF) of only approximately 3-4% [10]. Stem cell therapy has emerged as a promising strategy for the treatment of dead myocardium, directly or indirectly, and seems to offer functional benefits to patients [11].

Recently, a substantial number of clinical trials have proven that stem cell therapy is safe [12]. Infusion of bone marrow-derived stem cells (BMCs) represents the greatest number of clinical studies for MI. The overall efficacy for BMCs from meta-analysis on multiple published data has been inconsistent but relatively modest, with an improvement in LVEF of approximately 3-4% [11]. The majority of BMCs data for therapy, however, is less than ideal due to the limited component of active undifferentiated stem cells existing in bone marrow from early studies [13]. Many different types of stem cell with greater potential for cardiomyocyte regeneration, such as mesenchymal stem cells, cardiac stem cells, cardiosphere-derived cells, embryonic stem cells, and induced pluripotent stem cells, have been investigated in preclinical studies or clinical trials, which may help to improve the efficacy of cell therapies in cardiomyopathy [14]. The discrepancies among the multiple clinical studies may result from the various types of stem cells utilized in the studies as well as their different isolation and delivery procedures [15]. The beneficial outcomes from cell therapy are associated with paracrine effects, rather than direct regeneration of new tissue [5]. Therefore, large phase III clinical trials will be needed to confirm the salubrious effect of stem cell therapies in MI over placebo control. This review provides a comprehensive overview of treatment with stem-like cells in preclinical and clinical studies to assess the feasibility and efficacy of this novel therapeutic strategy in ischaemic cardiomyopathy.

## 2. Types of Stem Cells for Cardiac Cell Therapy

### 2.1. Skeletal Myoblasts

Skeletal myoblasts (SKMs) are precursors of satellite cells, which remain in a quiescent state under the basal membrane of muscle fibres [29]. Autologous transplantation of SKMs is conceptually alluring for heart regeneration because SKMs are easily procured during muscle biopsies, because they are highly proliferative after muscle injury, and especially because they are resistant to ischaemia and hypoxia [30]. In June 2000, intramyocardial administration of autologous SKMs derived from the thigh muscle into a patient with severe ischaemic heart failure during CABG established the first use of cell therapy in MI [31]. Several preclinical trials for the use of SKMs to promote cardiac repair in both small and large animal models demonstrated increased LVEF and resulted in a decrease of left ventricular (LV) remodelling without significant formation of new myocardial fibrosis [32–34].

SKMs specifically express myogenic transcription factors including MyoD, Myf5, and PAX7 [35]. Coculture of SKMs and neonatal cardiac myocytes in intercalated discs induced transdifferentiation into cardiomyocytes that expressed the cardiac-specific markers GATA4 and Nkx2.5 together with N-cadherin and connexin 43 [36, 37]. Despite the exciting finding that approximately 10% of the SKMs contracted in synchrony with adjacent cardiomyocytes in vitro, no studies have documented functional coupling of SKMs with the myocardium in vivo [38].

In contrast, SKMs remain skeletal myocytes in the heart and do not differentiate into cardiomyocytes, and infused SKMs fail to contract synchronously with the native myocardium, which results in a high risk of arrhythmias [39]. To date, the largest randomized, placebo-controlled, phase II SKM trial (the MAGIC trial) demonstrated that there was no significant improvement in regional or global LV function after SKM implantation and was discontinued prematurely [40]. Furthermore, the high number of adverse cardiac events secondary to myoblast injections decreased the further application of this treatment due to the availability of other types of stem cells [41].

### 2.2. Bone Marrow-Derived Stem Cells

In studies of sex-mismatched heart transplantations in humans, the Y chromosomes from the host cells were identified to colonize and differentiate in the implanted heart, which indicated the existence of mobile stem-like cells [42]. Under physiological conditions, there are bone marrow-derived stem cells (BMCs) in circulation. Therefore, the discovery of an additional origin of regenerated cardiomyocytes by fate-mapping implies that circulating BMCs continuously contribute to myocytes renewal in humans by means of cell fusion and transdifferentiation after injury [16, 43]. Sceptics, however, have questioned whether in vivo BMCs can meaningfully differentiate into all three main types of cardiac cells, which include myocytes, smooth muscle, and endothelial cells [44].

BMCs contain several different cell populations including haematopoietic stem cells (HSCs) [45, 46], mesenchymal stem cells (MSCs) [47–49], and endothelial progenitor cells (EPCs) [50, 51]. Therefore, the plasticity of BMCs to proliferate, migrate, and also differentiate into multilineage cell types may arise from the mixture of stem-like cells [52]. HSCs, for example, are defined as a rare population of cells that express CD31, CD34, CD45, CD133, and kinase insert domain receptor (KDR) and are believed to be able to derive all blood lineages and possibly transdifferentiate into cardiomyocytes [53].

Comparatively, a large number of unfractionated autologous cells, in terms of bone marrow mononuclear cells (BMMNCs), are relatively easy to obtain from the pelvic bones of patients [54]. In fact, clinical application could therefore be expedited with very limited convincing preclinical evidence. Only 4 months after the pioneer study of BMC-derived myocardial regeneration in mice [55], the first clinical treatment of a 46-year-old patient with autologous unfractionated BMMNCs for acute MI exhibited significant salubrious effects [56]. However, the outcomes from the initial trials of human myocardial therapy with BMMNCs were mild and controversial [55, 57–59].

The TOPCARE-AMI trial was one of the first randomized clinical trials of BMMNC therapy. Patients who were treated with intracoronary BMMNCs showed an approximately 11% improvement of LVEF at the five-year follow-up study [60]. The long-term beneficial outcomes from the BALANCE study has also proved that treatment with BMMNCs can increase LVEF to 10%, decrease infarction size, and improve quality of life [46]. Conversely, in the BOOST trial, a single intracoronary transfer of unfractionated BMMNCs in patients with acute AMI provided only short-term benefits (approximately 7% improvement in LVEF), which lasted no more than 6 months [61]. Similarly, the ASTAMI trial observed no effect on global LV function after intracoronary injection of autologous BMMNCs during a study follow-up period ranging from 24 hours to 3 years [17, 62–64]. The results from the TIME trial also demonstrated no recovery of global or regional LV performance and excluded the effect of BMMNC injection time at either 3 days or 7 days [65]. The MYSTAR study utilized a combined delivery through both an intramyocardial and intracoronary route and showed a mild improvement in LVEF of approximately 3.5% [66]. The REGENT trial used a selected population of CD34+ and CXCR4+ progenitor cells derived from autologous BMMNCs to treat patients with MI. LVEF was not improved in either the selected or unselected cell-treated group compared with patients without cell treatment. However, patients with severely damaged LVEF, whose baseline was less than 37%, displayed a trend in favour of both cell treatments [67]. To further emphasize this point, 191 patients with an LVEF of 35% or less due to ischaemic cardiomyopathy were enrolled in the STAR-heart trial. After 12 and 60 months, the BMMNC-treated patients exhibited a statistically significant improvement in LV performance compared with the baseline from the control group, which suggests that cell therapy can affect mortality in patients with chronic heart failure [68]. In other phase II/III trials, a statistically significant but modest and probably not clinically relevant enhancement in LVEF was observed in the REPAIR-AMI study [62]. In addition, no significant improvement in LV function was demonstrated at 4 months in the SWISS-AMI study [69]. To date, over 3000 patients have been treated with bone marrow-derived cells in numerous clinical trials all over the world [70]. Among these studies, a meta-analysis that included 2625 patients enrolled from 50 publications demonstrated that patients treated with BMMNCs showed a relatively moderate refinement in LVEF (~3.96%) and smaller infarct size (*～*−4.03%), LV end-systolic volume (*～*−8.91 mL), and LV end-diastolic volume (*～*−5.23 mL) [71]. Although the clinical significance of these studies needs to be further evaluated, the mortality, recurrence of MI, and rehospitalization for heart failure were significantly lower in the BMMNC-treated patients than in the control subjects. These favourable findings have catalysed the demand for additional large-scale trials [71, 72].

The current use of unfractionated BMMNCs is limited because the vast majority of isolated BMMNCs contain differentiated haematopoietic cells and very few stem cells. Only approximately 2–4% of HSCs and 0.01% of MSCs in BMMNCs can effectively be utilized for stem cell therapy [70]. In addition, the discrepancies in the results of the different trials may be partially ascribed to variations in the variables in each trial, such as the number of cells injected, the cell preparations, the delivery procedures, and even the baseline extent of LV dysfunction and geometry of the patients. Therefore, a good manufacturing practice (GMP) process is indispensable to warrant the production of a quality-controlled cell product and prevent contaminations of the end product [73]. With regard to safety, autologous BMCs are still the most frequently used cell type for the treatment of acute MI because among all the clinical trials that have been conducted there have been no observations of carcinogenesis, arrhythmias, or any other adverse effects [74].

### 2.3. Bone Marrow-Derived Mesenchymal Stem Cells

Bone marrow-derived mesenchymal stem cells (MSCs) are capable of differentiating into all the cells of mesodermal lineage, including osteogenic, chondrogenic, and adipogenic cells [75]. MSCs can be characterized primarily as CD105^+^ CD90^+^ cells, which will also express CD17, CD29, CD44, CD73, CD106, CD124, and CD166. Their surface antigens are absent of the haematopoietic markers CD14, CD31, CD34, CD45, and CD133. The aforementioned rare population of MSCs in bone marrow can be isolated by plastic adherence and subsequently cultured in vitro [13]. Intriguingly, MSCs lack major histocompatibility complex class II (MHC II) antigens and therefore can evade immune surveillance, which renders allogeneic applications plausible [76]. Under specific microenvironmental stimuli, MSCs can be induced to transdifferentiate into skeletal muscle and cardiac muscle and form functional cardiomyocytes in vivo [49]. In general, the manual preparation of autologous BMMNCs takes at least 4 hours [73]. Compared with autologous therapy, allogeneic human bone marrow-derived MSCs may provide an alternative off-the-shelf product to resolve the logistic, economic, and timing restrictions.

In preclinical study, human bone marrow-derived MSCs reduced myocardial infarctions and increased cardiac function and angiogenesis via intramyocardial transplantation in rat models of ischaemic cardiomyopathy [77–79].

Clinically, the safety and efficacy of the administration of proprietary allogeneic human MSCs (Prochymal) in patients with MI have been evaluated since 2005. All MSCs were isolated and expanded from a single donor and intravenously injected into the infarcted artery. LVEF was increased by approximately 6.7% over baseline at 6 months [80]. The intramyocardial implantation of autologous MSCs was studied in the PROMETHEUS trial. Six patients who were treated with the MSCs exhibited up to a 9.5% improvement in LVEF and a 47.5% reduction in scar mass [81]. To compare the safety and efficacy between autologous and allogeneic MSC therapies for ischaemic cardiomyopathy, a phase I/II randomized comparison (the POSEIDON-pilot trial) demonstrated relatively equal clinical improvements in terms of functional status and quality of life from both therapies. More importantly, the POSEIDON-pilot trial highlighted the potential of an inverse dose response, in which the clinical endpoints from a 20 million cells' injectant showed greater improvement and longer sustainability than injectant from 200 million cells, which suggests the importance of dosing thresholds in future clinical study design [82]. Therefore, a phase II study (the TRIDENT trial) is in progress to further estimate the dosage [83]. In addition to bone marrow-derived cells, transplantation of MSCs derived from the umbilical cord matrix was investigated in the WJ-MSC-AMI study. During an 18-month follow-up, global LVEF was significantly improved by approximately 5% compared with the placebo group, which suggests that the umbilical cord matrix is an alternative source for MSC treatment [84]. Due to a lack of patient participants and placebo-controlled studies among the early studies [80, 82], a recent report from a phase II, randomized, open-label and placebo-controlled study (the SEED-MSC trial) indicated a moderate (~5.9%) enhancement of LVEF from patients treated with autologous MSCs once compared with the placebo group [85]. In addition, there are several ongoing phase II/III trials that assess the efficacy of both autologous and allogeneic MSC therapy in patients with ischaemic cardiomyopathy [86, 87]. Analogous to BMC treatment, MSC therapy also displayed a feasible safety profile, including no ventricular arrhythmias or immunological side effects.

### 2.4. Adipose-Derived Mesenchymal Stem Cells

The reasonable supply of other noncardiac cell types promises an alternative candidate for regenerative therapeutic strategies for the treatment of ischaemic heart failure, including adipose-derived stem cells (ADSCs) [88]. ADSCs can be harvested from the adipose tissue of patients with minimal invasiveness and expanded in vitro more rapidly than bone marrow-derived MSCs [89]. As mentioned above, ADSCs have a similar origin as MSCs, which lack MHC class II antigens to prevent the rejection from engraftment into host tissues [90]. More importantly, ADSCs are able to differentiate into mesodermal lineages, cardiomyocytes [91], and endothelial cells [92] upon induction. ADSCs share a common expression profile as CD105^+^ CD90^+^ cells and have a CD49d^+^ CD106^−^ signature for discrimination [93].

In animal studies, the transplantation of ADSCs resulted in a significant improvement in LVEF and angiogenesis and a significant reduction in infarct area compared with BMMNCs [94]. Furthermore, cardiac functions were also enhanced in the infarcted rat hearts after ADSC engraftment, whereas the secretion of cardiac protective soluble factors was also proposed to induce cardiac function [95].

Several clinical trials were completed recently to explore the safety and feasibility of ADSCs in the treatment of patients with acute MI, such as the APOLLO trial [96], the CSCC_ASC trial [97], and the PRESIE trial [98]. Among these trials, the PRESIE study suggested that autologous injection of ADSCs did not mitigate scar size or increase LVEF but stabilized the scar size in patients with advanced ischaemic heart disease [98]. The preliminary data from the PRESIE study elicited the safety of ADSC treatment; however, additional results are expected from other trials.

### 2.5. Cardiac Stem Cells

Retrospectively, circulating marrow stem-like cells may home to the myocardium and contribute to cardiac homeostasis [42]. In 2000, Deisher described a population of small, replicating, nonadherent cells, which were isolated from the heart of adult p53-deficient mice, as “cardiac stem cells” because of their multipotency [99]. Subsequently, the existence of resident stem-like cells through cardiac development resulted in substantial progress in autologous stem cell therapy for ischaemic cardiomyopathy. Cardiac stem cells (CSCs), by definition, are self-renewing cells that are able to differentiate into a minimum of three cardiac cell lineages: myocytes, smooth muscle, and endothelial vascular cells [100]. CSCs or putative innate cardiac progenitor cells were isolated from independent groups including, c-kit^+^ cells [101], Sca-1^+^ cells [102], Islet-1^+^ cells [103], SSEA-1^+^ cells [104], PDGFR*α*^+^ cells [105], side population cells [106], and cardiosphere-derived cells [107]. In clinical studies, however, the physiological roles of those cardiac stem/progenitor cells in regeneration of ischaemic cardiomyopathy have not yet been fully investigated except c-kit^+^ cells and cardiosphere-derived cells [108].

In particular, c-kit^+^ cells were characterized by the expression of stem cell antigens c-kit (CD117), Sca-1, and MDR1 and are absent of the haematopoietic surface antigens CD31, CD34, CD45, CD133 [101]. Besides, these c-kit^+^ cells were identified to colonize at the yolk sack [109], whereas the ligand of the c-kit receptor (SCF) is expressed in the foetal and neonatal myocardium [110]. These findings emphasize that stem-like cells may reside in the heart after birth. Moreover, when injected into the ischaemic heart in rats, a population of c-kit^+^ Lin^−^ cells can reconstitute a functional myocardium [101]. These cells are negative for blood lineage markers (Lin) and haematopoietic markers (CD34 and CD45), which highlights that the cardiac stem-like cells are in the myocardium long enough to lose their blood cell lineages [101, 111]. Endogenous CSCs have been detected in the extremely low proportion of only 0.01% of cardiomyocytes [101], which explains the low turnover rate under physiological conditions. However, because CSCs are native to the heart and are specifically restricted for differentiation into cardiac lineages, they represent an ideal cell candidate for heart regenerative therapy after ischaemic insult [54].

A meta-analysis systematically analysed 80 preclinical studies including 1970 animals and verified the consistency of the beneficial effects of CSC therapy on MI. The overall effect of CSC treatment in small animals was an improvement in LVEF by approximately 12% compared with the placebo groups, whereas an approximately 5% improvement in LVEF was observed in large animals [112].

The SCIPIO study was the first phase I, randomized, open-label clinical trial to evaluate autologous c-kit^+^ CSCs in patients with ischaemic MI whose LVEF was lower than 40%. The CSCs were isolated from the right atrial appendage during CABG surgery. Ex vivo expanded 1 × 10^6^ c-kit^+^ CSCs were administered to 16 patients through intracoronary infusion approximately 4 months after CABG. Compared with the patients from the control group, cardiac MRI showed an increase in LVEF by approximately 8% and 12.3% at 4 months and 12 months after CSC injection, respectively. No evidence of tumour formation was observed after a 1-year follow-up. At the preclinical level, only 4–8% of transplanted CSCs colonized and persisted in the myocardium 1 year after infusion [113]; however, it was speculated that clinically 4–8% of transplanted CSCs would be insufficient to account for the functional and structural benefits of the CSC treatment in SCIPIO study since specimens of transplanted myocardium were difficult to acquire [114]. It was suspected that the major mechanism for the beneficial effects might be attributable to paracrine factors, which are released by the injected cells to modulate the proliferation of the host cardiac cells [113]. The CONCERT-HF trial is recruiting patients to investigate the safety and efficacy of autologous bone marrow-derived MSCs and c-kit^+^ CSCs both alone and in combination for the treatment of ischaemic cardiomyopathy [115].

### 2.6. Cardiosphere-Derived Cells

Human cardiac stem-like cells can migrate out of in vitro cultured human myocardial biopsies and form spheroids in suspension conditions. Those spherical clusters are termed cardiospheres (CSps) and are capable of self-renewal and are positive for the endothelial marker CD31 and cardiac progenitor cell markers such as c-kit, CD-34, Sca-1, and Nkx2.5 [107]. In fact, CSps are a heterogeneous mixture of cardiac stem cells, differentiating progenitors and differentiated cardiomyocytes, depending on the size of the spheroid and the time in culture. C-kit^+^ cells were found to be localized in the centre of the spheroids and are positive for BrdU staining, which suggests the proliferation and differentiation hierarchy of c-kit+ cells in the growth of CSps [107]. Notably, cardiosphere-derived cells (CDCs) are able to differentiate into cardiomyocytes and vascular cells, and only the cells in the centre are maintained in an undifferentiated state, whereas the cells at the surface layer are continuously undergoing differentiation [116]. In addition, cardiac stem-like cells, similar to other types of adult stem cells, sustain their multipotency within an appropriate niche [117]. CDCs can also enhance cardiac function once injected into infarcted rat hearts [107].

CDCs exhibited superior cardiomyogenic differentiation potential, angiogenic formation, and paracrine factor secretion compared with BMMNCs, bone marrow-derived MSCs, and adipose-derived MSCs in mice [118]. Furthermore, a mixture of CDCs also outperformed purified c-kit^+^ CSCs in the same study, which suggests that the supporting cells somehow improve the function of the stem-like cells in vivo [118].

These results led to the initiation of several phase I clinical trials involving CSps, including the CADUCEUS [119] and ALCADIA [120] trials, which assessed the feasibility and safety of an intracoronary injection of autologous CSps after a recent infarct. Results from the CADUCEUS study showed an average decreased scar size of 12.3% at 12 months. Although regional function was improved, no improvement in global function was reported [121]. Safety concerns were raised during the injection of CSps because their size (50–200 *μ*m) may potentially cause capillary plugging [122]. Larger studies to evaluate the safe dosage and efficacy of these treatments are in demand.

As with MSCs, CDCs exhibit mesenchymal properties and lack MHC II antigen, which endow the application of allogeneic CDCs [123]. The safety and efficacy between autologous and allogeneic CDCs transplantations to treat ischaemic cardiomyopathy in large animal studies were comparable [124], resulting in at least three ongoing clinical trials, including ALLSTAR, DYNAMIC, and HOPE, for further investigations [125–127].

### 2.7. Embryonic Stem Cells and Induced Pluripotent Stem Cells

Embryonic stem cells (ESCs) are derived from the inner cell mass of the early embryo in the blastocyst stage. They are self-renewing, clonogenic, and capable of differentiating into any type of cell in the adult, including cardiomyocytes [128, 129]. It has been proven that ESCs are able to differentiate into all specialized cell types in the heart in vitro, such as atrial-like, ventricular-like, sinus nodal-like, and Purkinje-like cells [130]. The expression of cardiac-specific transcription factors such as GATA4, Nkx2.5, Mef2c, and Irx4 has been found in human ESC-derived cardiac cells [131]. Moreover, cultured ESC-derived cardiomyocytes beat spontaneously and synchronously under physiological conditions [132]. In a mouse model of myocardial infarction, direct injection of 5 × 10^4^ genetically engineered ESCs improved cardiac function by 4 weeks. From that study, 21% of the mice formed teratomas after transplantation because of the unlimited differentiation potential of ESCs [133]. Therefore, highly enriched cardiomyocytes derived from murine ESCs, which were produced through the selection of the cardiac-specific promoter NCX1 and constructed with puromycin-resistant cells from embryonic bodies, circumvented the issue of teratoma formation [134].

However, embryonic stem cells may not be ideal for clinical application because of their ethical concerns [135], potential genetic instability [133], and the risk of immune rejection [136]. Currently, only one clinical trial is actively recruiting patients to test the use of human embryonic stem cell-derived CD15^+^ Isl-1^+^ progenitors in severe heart failure (the ESCORT study) [137].

Enforcing expression of OCT4, SOX2, KLF4, and c-MYC transcription factors can reprogram terminally differentiated cells to closely resemble embryonic stem cells, which are termed induced pluripotent stem cells (iPSCs) [138]. Functional cardiomyocytes have now been successfully derived from both mouse [139] and human iPSCs [140]. As an alternative source for all cardiogenic cell lines, iPSCs can be derived from individual patients for autologous transplantation to minimize the risk of immune rejection and resolve the ethical issues [138].

The pitfalls of iPSC application include the risk of teratoma formation associated with the pluripotent state, whereas defining reliable methods for inducing highly enriched populations of cardiomyocytes are essential [141–143]. Meanwhile, the low efficiency of cardiogenic differentiation, high costs, and time-consuming methods (approximately 4 months) of iPSCs require further investigation [144], which should also explore the generation of cardiomyocytes from somatic cells without transit through a pluripotent state [145, 146]. Attempts to manufacture clinical grade iPSCs products from blood and skin samples are in progress [147, 148].

## 3. Clinical Indications and Unresolved Issues

As mentioned above, the vast majority of completed human clinical trials are difficult to clarify and compare because the delivered cells are either mixed or enriched populations, and the number of implanted cells, delivery methods, and injection time intervals after MI are different (Table 1). Taken together, clinical endpoints from stem cell treatment in MI are feasible and safe; however, their efficacy has been inconsistent but modest, which allows significant room for improvement.

The variable and moderate benefits associated with stem cell treatments were initially ascribed to inefficient cell delivery, with only 10% (or less) of the cells retained in the heart after 24 hours regardless of the cell type or delivery route [149]. The cells are usually washed out through the coronary venous system or mechanically ejected from the injection site [150]. Repeated administrations of BMCs, MSCs, and CDCs were demonstrated to boost therapeutic benefits for chronic heart failure [151–153]. The following are three routes for delivering stem cells for cardiac therapy: systemic intravenous infusion, intracoronary infusion, and intramyocardial injection. For intravenous infusion, only 0.04% of the infused cells reach the infarct region, whereas uptake of cells was found in other organs, especially in the lungs [154]. The advantage to intravenous injection is its simplicity and least invasive nature, which allows the option of treatment with repeated cell injections. Its safety and feasibility have been verified in a phase I clinical trial with a 1-year follow-up [80]. And the efficacy for intravenous injection of human MSCs after AMI was conducted under a phase II study [155].

Direct intramyocardial injection through open-chest surgery during CABG offers the most precise and accurate approach for implanting stem cells into the infarcted region of the heart [156]. But the invasive nature of this operation increased the risk of complications and mortality and prolonged the period for recovery. To circumvent the demand of delivery routines for surgically high-risk patients and applications for repeated therapies, percutaneous catheter-based intramyocardial injection has been developed dependent on the arterial access of individual patient, such as transcoronary venous approach [157] and transendocardial approach [58]. In a substudy of the MYSTAR trial, the myocardial perfusion imaging with single photon emission computer tomography (SPECT) unraveled an average increase in tracer uptake of 6.2% BMCs in intramyocardial area, suggesting a major beneficial effect on those patients exhibiting individual improvement via intramyocardial injection [158].

For the most clinically practiced procedure, intracoronary delivery uses balloon catheters to infuse stem cells into a coronary artery, where blood flow was interrupted by inflating the balloon to homogenously distribute stem cells [159]. There is still substantial loss of injected cells due to extravasated and venous washout [160]. Tracing BMCs labelled with 18-fluorodeoxyglucose revealed that only 1.3% to 2.6% of cells were retained in the infarcted myocardium after intracoronary transplantation [161]. Treatment efficacy was increased through physical retention of the cells with fibrin glue [150] and biomaterial scaffolds [162, 163] to boost homing of the stem cells in the host tissue.

Indeed, a remarkable 6700-fold range in cell dosage was nonsystematically investigated through the vast majority of preclinical and clinical studies worldwide [164]. It was suggested that there might be a threshold between the dosage of BMCs and the therapeutic effects [72].

Due to inflammation or anoikis, over 90% of the successfully retained cells are not able to survive after one week [165], of which less than 1% of the infused cells can be identified 4 weeks after transplantation [166]. Most stem-like cells die within days or weeks of transplantation into infarcts [167], although discrepancies may also arise from different injection times among or even within each clinical trial or in variations in the techniques for cell isolation, incubation, and expansion. Compared with unfractionated BMMNCs, the preparation and expansion of bone marrow-derived MSCs for therapy require at least 7 days, whereas the endogenous conditions from each patient may be varied [168]. Sufficient time for the selection and expansion of specific progenitor cells and particularly allogeneic off-the-shelf cell products is urgently needed.

The pathological conditions for acute infarction, chronic ischaemia, and chronic heart failure are distinctive in regard to each patient's local vascular, cellular, and chemical microenvironments. Patients who suffer from worse baseline myocardial infarctions seem to benefit the most from cell therapy in several clinical scenarios. In the BOOST study, functional improvement was observed only in cell-treated patients with greater infarcted areas [61]. Similarly, in the REGENT and REPAIR-AMI studies, only patients with lower baseline LVEF showed sustained recovery at a later time point [67, 169]. These findings indicate that future design of studies for cell therapy should target a sicker patient population [54]. Though enrolment of patients with poorer LVEF may favour their risk-benefit ratio [170], cardiovascular risk factors, such as age, hypertension, diabetes, hyperlipidemia, smoking, obesity, and hyposthenia, all need to be considered in clinical studies. These cardiovascular risk factors were reported to negatively influence the proliferation and function of stem cells, which will indeed compromise the efficacy of autologous cell therapy in pilot studies [171]. For instance, progenitor cells from the elderly showed decreased telomerase activity and increased cellular senescence, suggesting an age-related decline of outcomes from marrow-derived cell treatment [172]. Diabetes not only impairs the number of BMCs but also interferes with the homing signals to prevent vascular integration, leading to reduced benefits from cell therapy [173, 174]. In BONAMI trial, active smokers showed impairments in cardiac function recovery from BMCs treated group due to mobilization of progenitor cells [175]. It was suggested that LVEF can be profoundly ameliorated by BMCs transplantation combined with CABG and early onset (7–14 days after MI) engraftment [176].

To date, tissue retention of implanted stem-like cells is disappointingly low; however, their salubrious effects can last for years, which suggests indirect paracrine effects that activate endogenous regenerative mechanisms to benefit the infarcted hearts [177]. Despite the extremely low prevalence of HSCs in initial unfractionated BMMNC therapy with improvement in LVEF, subsequent studies have argued that HSCs did not directly differentiate into cardiomyocytes but instead became mature blood lineage cells after transplantation [45, 178]. In particular, the administration of cell culture medium conditioned by MSCs overexpressing the gene AKT-1 significantly reduced infarct size and cardiac apoptosis [179]. Cardiac protective growth factors, such as vascular endothelial growth factor (VEGF), hepatocyte growth factor (HGF), and insulin-like growth factor 1 (IGF-1), were also found in ADSC culture medium [95]. Nevertheless, evidence from genetic fate-mapping suggested that c-kit^+^ CSCs and CDCs promote cardiomyocyte renewal after infarction without direct differentiation into cardiomyocytes in mice [113, 180, 181]. Collectively, the transplanted cells are proposed to produce soluble factors that can reduce scar formation and therefore ameliorate the niche for cardiomyocyte growth. [177]. Further identification of paracrine effectors may allow the development of defined, cell-free treatments based on proteins or small molecules [182]. Putative paracrine factors released by stem-like cells were listed in Table 2.

Emerging experience has focused on unmodified derivations of adult stem cells, whereas optimization of paracrine profile of those implanted stem cells may regulate their therapeutic effects on injured myocardium [183]. For instance, granulocyte colony-stimulating factor (G-CSF) can accelerate healing process of cardiomyocyte regeneration via mobilization of endogenous BMCs into peripheral blood [184]. The relative efficacy of combination of G-CSF and BMCs administration was shown to be promising in several clinical studies [185, 186]. Activation of stromal derived factor-1 (SDF-1), which contributes to myocardial homing of c-kit^+^ CSCs, thereby may further improve the efficacious outcomes when combined with G-CSF treatment [187]. Alternatively, enhanced expression of IGF-1 in CSCs was shown to boost paracrine mediated regenerative capacity in infarcted myocardium by promoting transplanted cell proliferation and survival [183]. Based on that, predifferentiation of adult stem cells into cells with cardiopoietic phenotype may enhance their survival and engraftment during myocardial implantation [188]. A proof-of-concept study has thereby established a recombinant cardiogenic cocktail consisting of TGF*β*1, BMP-4, activin-A, retinoic acid, IGF-1, FGF-2, *α*-thrombin, and IL-6 to guide human MSCs into reparative cardiopoietic progenitor in a murine model [189].

## 4. Future Prospects

The legacy of these preclinical and clinical findings has promoted a consensus about the criteria by which regenerative stem cell therapies are assessed. The ideal candidate donor cell for myocardial reconstitution is a stem-like cell that can be easily obtained, has a robust proliferation capacity and a low risk of tumour formation and immune rejections, differentiates into functionally normal cardiomyocytes, and is suitable for minimally invasive clinical routines for transplantation. The use of stem cell therapy for heart disease is a complicated and still poorly understood process and requires a standard protocol for the characterization and quality control of stem cell preparation and comparable methodologies for cell delivery, dosage, timing, and clinical patient selection. The considerable advances in our current understanding have shown that stem cell therapy is safe, is moderately effective, and is mediated by indirect paracrine mechanisms. Dissection of the paracrine effectors induced by stem-like cells in cardiac regeneration will also pave the way for therapeutic interventions.

## Figures and Tables

**Table 1:** Published stem-like cell clinical trials for cardiac myopathy.

Study name (sponsor)	NCT ID	Number of patients (treatment/controls)	Delivery route	Cell dose (×10^6^)	Follow-up interval (months)	Outcomes	Adverse effect
*Skeletal myoblasts*							
MAGIC (Genzyme)	NCT00102128	67/30	IM	Low dose: 400 High dose: 800	6	Neutral for LVEF Reduced LVESV (−9.5%) Reduced LVEDV from high dose (−13.6%)	Low dose: 4 with ventricular arrhythmias and 5 deaths High dose: 5 with arrhythmias and 4 deaths
CAUSMIC (Mytogen)	NCT00626314	12/11	IM	30, 100, 300, 600	12	Improved LVEF (12.4%) Reduced NYHA	6 with ventricular arrhythmias out of 12 patients
SEISMIC (Bioheart)	NCT00375817	26/14	IM	150–800	6	Neutral for LVEF Reduced for NYHA Increased 6 MWD	12 with ventricular arrhythmias out of 26 patients and 1 death
*Bone marrow mononuclear cells*							
Janssens et al. (Universitaire Ziekenhuizen Leuven)	NCT00264316	33/34	IC	304	4	Neutral for LVEF Reduced infarct size (−28%)	No major adverse events
REPAIR-AMI (A. M. Zeiher)	NCT00279175	102/102	IC	200	24	Improved LVEF (2%) Reduced LVESV (−1%)	No major adverse events
Cardio 133 (German Heart Institute)	NCT00462774	30/30	IM	5	6	Reduced LVEF (−2.1%) Neutral LVESV Neutral LVEDV	No major adverse events
FINCELL (University of Oulu)	NCT00363324	40/40	IC	360	6	Improved LVEF (5%) Reduced LVESV (−5.7%) Reduced LVEDV (−1.3%)	No major adverse events
MiHeart-AMI (Ministry of Health, Brazil)	NCT00350766	150/150	IC	100	12	No study results	No study results
BOOST (Hannover Medical School)	NCT00224536	30/30	IC	2000	6, 18, 60	Larger scar: improved LVEF (7.6%) Smaller scar: reduced LVEF (−6.6%) Increased LVESV (42%) Increased LVEDV (29%)	No major adverse events
DanCell-CHF (Odense University Hospital)	NCT00235417	32/0	IC	1st: 650 2nd: 900	4, 8, 12	Neutral for LVEF Improved LV filling	No major adverse events
MYSTAR (Medical University of Vienna)	NCT00384982	40/20	IC/IM	200	3	Early group: Improved LVEF (3.5%) Late group: Improved LVEF (3.9%)	No major adverse events
BALANCE (University of Düsseldorf)	Reference [46]	62/62	IC	60	3, 12, 60	Improved LVEF (10%) Reduced LVESV (−4.0%) Reduced infarct size (−8.2%)	No major adverse events
REGENT (Silesian School of Medicine)	NCT00316381	160/40	IC	200	6	Neutral for LVEF	No major adverse events
STAR-heart (University of Düsseldorf)	Reference [68]	191/200	IC	66	3, 12, 60	Improved LVEF (6.2%) Reduced LVESV (−12.9%) Reduced LVEDV (−5.1%)	No major adverse events
AMRS (Emory University)	NCT00313339	28/22	IC	5, 10, 15	6	Improved LVEF (2.1%) Reduced infarct size (−5.5%)	No major adverse events
TOPCARE-AMI (Johann Wolfgang Goethe University Hospital)	NCT00289822	29/30	IC	5.5	60	Improved LVEF (11.6%) Reduced LVESV (−16.3%) Reduced LVEDV (−49.5%)	No major adverse events
Hu et al. (China National Center for Cardiovascular Diseases)	NCT00395811	31/29	IC	10–700	6	Improved LVEF (5.7%) Reduced LVESV (−14.8%) Reduced LVEDV (−10.7%)	No major adverse events
BONAMI (Nantes University Hospital)	NCT00200707	52/49	IC	100	12	Neutral for LVEF Neutral for infarct size	1 death in cell treatment group after 1 month
PERFECT (Miltenyi Biotec GmbH)	NCT00950274	41/40	IM	0.5–5	6	No study results	No study results
TIME (The University of Texas Health Science Center, Houston)	NCT00684021	43/24	IC	150	6	Neutral for LVEF	No major adverse events
ASTAMI (Oslo University Hospital)	NCT00199823	50/50	IC	80	3, 6, 12, 36	Neutral for LVEF Reduced infarct size (−25%)	No major adverse events
SCAMI (University of Ulm)	NCT00669227	28/14	IC	324	6, 12, 24, 36	6 months: improved LVEF (4.1%) 12 months: improved LVEF (3.8%) 24 months: improved LVEF (4.5%) 36 months: reduced LVEF (5.6%)	No major adverse events
METHOD (Cardiocentro Ticino)	NCT01666132	36/18	IC/IM	50–500	6, 12	Neutral for LVEF	No major adverse events
SWISS-AMI (University of Zurich)	NCT00355186	128/64	IC	119–153	4, 6, 12	Neutral for LVEF Reduced LVESV (−9.4%) Reduced LVEDV (−8.4%) Reduced infarct size (−4.67%)	No major adverse events
TAC-HFT (University of Miami)	NCT00768066	19/10	IM	100, 200	12	Neutral for infarct size	No major adverse events
Helsinki BMMC (Helsinki University)	NCT00418418	20/19	IC	840	12	Neutral for LVEF Reduced infarct size (13.1%)	No major adverse events
MultiStem (Athersys, Inc.)	NCT00677222	19/6	IC	20, 50, 100	4	20 M cells: Improved LVEF (3.9%) 50 M cells: Improved LVEF (13.5%) 100 M cells: Improved LVEF (10.9%)	No major adverse events
Hu et al. (Second Affiliated Hospital, School of Medicine, Zhejiang University)	NCT01234181	22/14	IC	100	6, 12	Neutral for LVEF	No major adverse events
Ge et al. (Fudan University)	NCT02425358	79/25	IC	500	12	24 after PCI: improved LVEF (4.5%) 3−7 days after PCI: improved LVEF (3.5%) 3−30 days after PCI: improved LVEF (1.3%)	No major adverse events
TECAM (TECAM Group)	NCT00984178	89/31	IC	5	12	Improved LVEF (4%) Neutral for LVESV Neutral LVEDV	No major adverse events
REGENERATE-AMI (Barts & The London NHS Trust)	NCT00765453	55/45	IC	59.8	3, 12	Improved LVEF (2.2%) Reduced infarct size (−33.3%)	No major adverse events
							
*Mesenchymal stem cells*							
Prochymal (Mesoblast International Sàrl)	NCT00114452	39/21	IV	30–300	3, 6, 12	Improved LVEF (6.7%)	No major adverse events
POSEIDON-Pilot (University of Miami)	NCT01087996	15/16 (autologous/allogeneic)	IM	20, 100, 200	13	Neutral for LVEF 20 M cells: Improved LVEF Reduced infarct size (−33.21%) Allogeneic: Reduced LVEDV	No major adverse events
PROMETHEUS (Joshua M Hare)	NCT00587990	6/3	IM	20, 200	3, 6, 18	Improved LVEF (9.5%) Reduced infarct size (−47.5%)	No major adverse events
TAC-HFT (University of Miami)	NCT00768066	19/10	IM	100, 200	12	Neutral for LVEF Reduced infarct size (−18.9%) Increased 6 MWD	No major adverse events
SEED-MSC (Yonsei University)	NCT01392105	30/28	IC	72	6	Improved LVEF (5.9%)	No major adverse events
Stempeucel (Stempeutics Research Pvt. Ltd.)	NCT00883727	10/10	IV	200	24	Improved LVEF (2%)	No major adverse events
WJ-MSC-AMI (Navy General Hospital, Beijing)	NCT01291329	58/57	IC	6	18	Improved LVEF (5%) Reduced LVESV (−22.6%) Reduced LVEDV (−9.5%)	No major adverse events
							
*Adipose derived mesenchymal stem cells*							
PRECISE (Cytori Therapeutics)	NCT00426868	21/6	IM	42	6, 12, 18	Neutral for LVEF Reduced infarct size (−12.8%)	No major adverse events
CSCC_ASC (Jkastrup, Denmark)	NCT02387723	10	IM	100	6	No study results	No study results
ATHENA (Cytori Therapeutics)	NCT01556022	14/14	IM	40	6, 12	No study results	No study results
							
*Cardiac stem cells*							
SCIPIO (University of Louisville)	NCT00474461	16/7	IC	1	4, 12	Improved LVEF (12.3%) Reduced infarct size (−30.2% )	No major adverse events
CADUCEUS (Cedars-Sinai Medical Center)	NCT00893360	17/8	IC	12.5–25	6, 12	Neutral for LVEF Neutral for LVESV Neutral for LVEDV Reduced infarct size (−12.3%)	4 patients had serious adverse events

IC, intracoronary infusion; IM, intramyocardial injection; IV, intravenous infusion; LV, left ventricular; LVEDV, LV end-diastolic volume; LVEF, LV ejection fraction; LVESV, LV end-systolic volume; MWD, minute walk distance; NYHA, New York Heart Association; PCI, percutaneous coronary intervention.

**Table 2:** Putative paracrine factors released by stem-like cells.

Putative paracrine factor	Symbol	Proposed function	Reference
ABI family member 3 binding protein	ABI3BP	Cell development; tissue remodeling	[18]
Adipocyte complement-related protein	ADIPOQ	Cell growth; angiogenesis; tissue remodeling	[19]
Angiopoietin 1	AGPT 1	Angiogenesis	[20, 21]
Angiopoietin 2	AGPT 2	Angiogenesis	[20]
Agouti-related protein	AgRP	Homeostasis	[19]
Angiogenin	ANG	Angiogenesis; proliferation	[18, 19]
Bone morphogenetic protein 2	BMP2	Cell development	[19, 20]
Bone morphogenetic protein 4	BMP4	Cell development; differentiation	[20]
Chemokine ligand 23	CCL23	Cytoprotection; cell proliferation	[19]
Colony stimulating factor	CSF1	Cell proliferation; differentiation	[20]
Chemokine ligand 13	CXCL13	Inflammation; cell development	[19]
Fibrillin 1	FBN1	Structural protein; cell signal	[19]
Fibroblast growth factor 1	FGF1	Cell proliferation; migration	[18–20, 22]
Fibroblast growth factor 2	FGF2	Cell proliferation; migration	[20]
Fibroblast growth factor 6	FGF6	Cell proliferation; angiogenesis; differentiation	[19]
Fibroblast growth factor 7	FGF7	Cell proliferation	[19]
Hypoxic-induced Akt regulated stem cell factor	HASF	Cytoprotection; cell proliferation	[18]
Hepatocyte growth factor	HGF	Cell migration; angiogenesis; cytoprotection	[18, 20, 22]
Insulin-like growth factor 1	IGF1	Cell growth; proliferation; cytoprotection	[18, 20, 23, 24]
Interleukin 1	IL1	Inflammation; cell signal	[20]
Interleukin 2	IL2	Inflammation; cytoprotection; proliferation; differentiation	[25]
Interleukin 5	IL5	Inflammation; cytoprotection; proliferation; differentiation	[19]
Interleukin 6	IL6	Inflammation; cell signal	[20, 22, 25]
Interleukin 8	IL8	Inflammation	[25]
Interleukin 10	IL10	Inflammation; cell signal	[19]
Interleukin 12B	IL12B	Inflammation; cytoprotection; cell growth	[25]
Interleukin 16	IL16	Inflammation; proliferation	[25]
Inhibin beta A	INHBA	Cell signal; cell growth	[19]
Integrin *β*1	ITG*β*1	Cell signal; cell attachment	[20]
MicroRNA 132	miR-132	Inflammation; angiogenesis; cell development	[26]
MicroRNA 146a	miR-146a	Cell growth; proliferation	[26, 27]
MicroRNA 210	miR-210	Angiogenesis; cytoprotection	[26]
Matrix metalloproteinase 2	MMP2	Extracellular matrix degradation	[20, 28]
Matrix metalloproteinase 3	MMP3	Extracellular matrix degradation	[25]
Matrix metalloproteinase 9	MMP9	Extracellular matrix degradation	[20, 28]
Matrix metalloproteinase 27	MMP27	Extracellular matrix degradation	[19]
Nerve growth factor	NGF	Cytoprotection	[20]
Neuregulin	NRG	Angiogenesis; cell proliferation	[18]
Netrin G1	NTNG1	Cell development	[19]
Orosomucoid 2	ORM2	Inflammation	[19]
Platelet-derived growth factor	PDGF	Cell proliferation; angiogenesis	[18, 20]
Prostaglandin E2	PGE2	Cell development; cell proliferation	[18]
Periostin	POSTN	Cell proliferation	[18]
Resistin	RETN	Cell signal	[19]
Stromal derived factor	SDF	Cell development; angiogenesis	[18, 22]
Secreted frizzled related protein 1	SFRP1	Cell development	[19]
Secreted frizzled related protein 2	SFRP2	Cell development; tissue remodeling; cytoprotection	[18]
Transforming growth factor *β*	TGF*β*	Angiogenesis; cell proliferation	[18, 20, 24]
Tissue inhibitor of metalloproteinase	TIMP	Cell migration; extracellular matrix degradation	[20]
Tumor necrosis factor	TNF	Cell proliferation; extracellular matrix degradation	[20]
Vascular endothelial growth factor	VEGF	Angiogenesis; cytoprotection; proliferation	[18, 20, 24]
Von Willebrand factor	VWF	Cytoprotection	[19]
